# Neuronal polarization in the developing cerebral cortex

**DOI:** 10.3389/fnins.2015.00116

**Published:** 2015-04-08

**Authors:** Akira Sakakibara, Yumiko Hatanaka

**Affiliations:** ^1^College of Life and Health Sciences, Chubu UniversityKasugai, Japan; ^2^Division of Cerebral Circuitry, National Institute for Physiological SciencesOkazaki, Japan; ^3^Japan Science and Technology Agency, Core Research for Evolutional Science and TechnologyTokyo, Japan

**Keywords:** neuron, polarization, axon, cerebral cortex, imaging, excitatory cortical neuron, inhibitory cortical neuron

## Abstract

Cortical neurons consist of excitatory projection neurons and inhibitory GABAergic interneurons, whose connections construct highly organized neuronal circuits that control higher order information processing. Recent progress in live imaging has allowed us to examine how these neurons differentiate during development *in vivo* or in *in vivo*-like conditions. These analyses have revealed how the initial steps of polarization, in which neurons establish an axon, occur. Interestingly, both excitatory and inhibitory cortical neurons establish neuronal polarity *de novo* by undergoing a multipolar stage reminiscent of the manner in which polarity formation occurs in hippocampal neurons in dissociated culture. In this review, we focus on polarity formation in cortical neurons and describe their typical morphology and dynamic behavior during the polarization period. We also discuss cellular and molecular mechanisms underlying polarization, with reference to polarity formation in dissociated hippocampal neurons *in vitro*.

## Introduction

Neurons are highly polarized cells that typically exhibit a single axon and several dendrites. Dendrites receive incoming signals at the synapse and convey them to the soma. These signals trigger action potentials at the level of soma, which propagate along the axon and are transmitted to target cells at a presynaptic site. A critical question in neurobiology is how neurons acquire axon-dendrite polarity, a property required for directional information flow in the nervous system.

Axon-dendrite polarization has been historically examined using cultured, dissociated hippocampal neurons (Dotti et al., 1988). These neurons initially appear symmetric, extending and retracting several immature neurites of similar length. Elongation of a single process, the one that will become the axon, breaks this symmetry. Thus, based on this model, neuronal polarity formation has been believed to result from a stochastic symmetry-breaking event. However, more recent morphological and imaging studies *in vivo* or *in situ* (in *in vivo*-like conditions that maintain an intact three-dimensional structure surrounding immature neurons) suggest that several types of neurons establish neuronal polarity by inheriting apicobasal polarity from neuroepithelial progenitors or maintaining front-rear polarity in migrating cells (Barnes and Polleux, 2009; Hatanaka et al., 2012). Therefore, it remained uncertain whether these activities occurred *in vivo* and, if so, whether they were regulated by similar events as that appear in cultured hippocampal neurons.

The cerebral cortex is evolutionary the youngest and the most complex region of the brain. It is composed primarily of excitatory neurons, which are glutamatergic, and by a smaller proportion of inhibitory neurons, which are GABA (γ-aminobutyric acid)-ergic. During development, excitatory neurons originate from the dorsal telencephalon (Molyneaux et al., 2007), while inhibitory neurons originate from the ventral telencephalon (Gelman and Marin, 2010). Both subtypes are then integrated into the cerebral cortex and extend axons and dendrites to establish functional cortical circuitry. Interestingly, recent imaging studies have revealed how these dynamic developmental processes occur *in vivo* or *in situ*. Those studies suggest that most cortical neurons likely establish an axon via an initial symmetry-breaking event, a process similar to that observed in cultured hippocampal neurons.

In this review, we first give an overview of current knowledge about the developmental process of axon and dendrite formation of excitatory and inhibitory neurons in the cerebral cortex. Then we focus on the dynamic behavior underlying axon formation of these neurons, with reference to dissociated hippocampal neurons. Although there is evidence that some neurons may inherit some aspects of polarity emerged at a stage prior to axon formation, the model based on hippocampal cells still predominates in this field and could explain behavior of cortical neurons. We thus further summarize both intracellular signals and cytoskeletal dynamics underlying polarity formation in dissociated hippocampal neurons and in cortical neurons *in vivo* or *in situ*.

## Axon-dendrite polarization of excitatory and inhibitory cortical neurons

The cerebral cortex is composed of the neocortex and allocortex. The neocortex, which is a six-layered structure unique to mammals, is phylogenetically the youngest brain region and comprises most of the cortex. In contrast, the allocortex is phylogenenetically older and characterized by fewer layers than the neocortex. The development of polarity by neocortical cells is the major focus of this review.

In rodents, cortical neurons are comprised of 70–80% excitatory and 20–30% inhibitory neurons. Excitatory projecting neurons convey cortical output to subcortical structures and to other cortical areas. In general, neurons exhibiting corticofugal projections, which extend axons away from the cortex, reside in deep layers; by contrast, neurons that project intracortically extend axons to areas in the ipsilateral and/or contralateral cortex, reside in upper layers and to a lesser extent in deep layers (Greig et al., 2013). Depending on layer location and projection, excitatory neuron morphology varies. However, many excitatory neurons resemble so-called “pyramidal cells”: their soma is shaped like a pyramid with a base facing the apical aspect of the cortex, and these cells extend a single axon and two separate apical and basal dendrites (Jones, 1984). Their axons extend toward the white matter (WM) where they typically turn and continue to project tangentially, while their apical dendrites extend toward the pial surface. Inhibitory neurons, on the other hand, are mostly local-circuit neurons that contribute to intracortical information processing by modulating excitability and thus shaping cortical output. Inhibitory neurons also extend a single axon and multiple dendrites, but their morphologies are highly diverse: they include basket cells, chandelier cells, Martinotti cells, double bouquet cells, neurogliaform cells, and at least 10 others (Kubota, 2014). Until now, however, only a few reports have described how axons or dendrites emerge from these neurons (e.g., Kawaguchi, 1993).

## Axon formation is the initial step of cortical neuronal polarization

Excitatory and inhibitory cortical neurons originate in distinct brain regions: the former emerge from the pallium and the latter primarily from the subpallium (Molyneaux et al., 2007; Gelman and Marin, 2010). Recent advances in cell labeling techniques, including use of genetically-modified mice and *in utero* electroporation methods, allow us to label these neurons accurately. Furthermore, advanced imaging techniques have revealed dynamic processes underlying their development.

### Development of excitatory cortical neurons

**Figure 1:**
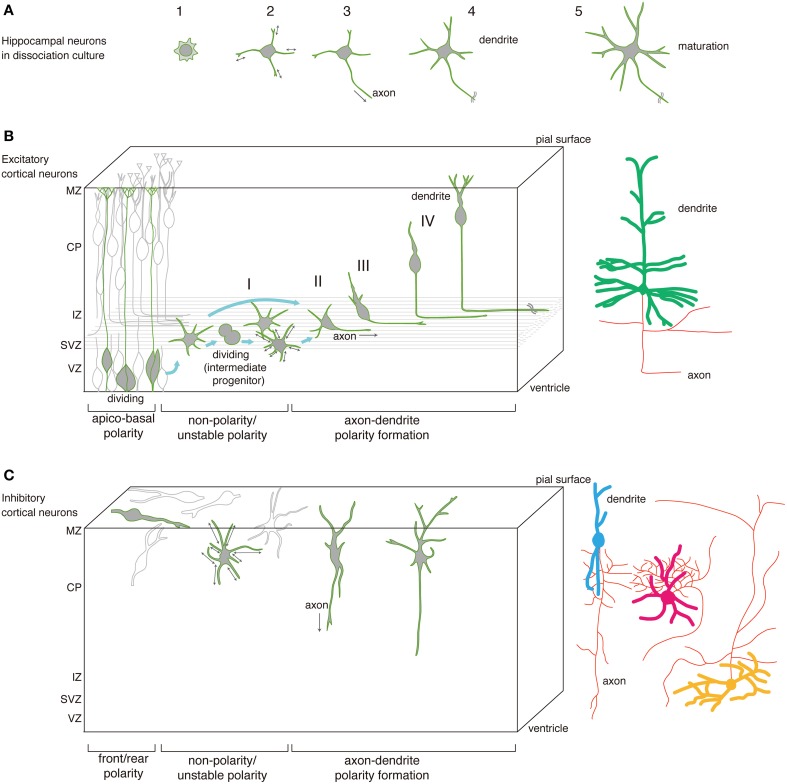
**Sequential events of polarity formation as seen in hippocampal neurons *in vitro* and excitatory and inhibitory cortical neurons *in vivo***. Axon outgrowth processes are similar *in vivo* and *in vitro*, as axons emerge from non-polarized cells. **(A)** Schematic drawing showing neuronal polarity formation in dissociated hippocampal neurons in culture. (1) Immature neurons actively form filopodia and lamellipodia and then (2) extend multiple minor processes that randomly extend and retract their tips. (3) After several hours in culture, a minor process begins to grow rapidly and transform into an axon (symmetry break). (4) That axon further extends, and remaining processes differentiate into dendrites. (5) Finally, these differentiated processes mature. **(B)** Schematic drawing showing acquisition of neuronal polarity by excitatory cortical neurons. (I) Young neurons differentiated from ventricular zone (VZ) cells or through intermediate progenitors transform into multipolar cells, whose short processes repeatedly extend and retract in the subventricular zone (SVZ)/intermediate zone (IZ) over several hours. (II) A new process, which will become the axon (symmetry break), suddenly elongates tangentially. (III) The remaining processes, which will become dendrites, transform into a pia-directed leading process. (IV) Neurons gradually change shape, become bipolar, and migrate radially toward the pia, with the elongating axon as the trailing process. After reaching their final destination, axonal and dendritic processes mature. Most excitatory neurons differentiate into pyramidal cells (dendrites, green; axons, red). **(C)** Schematic drawing showing neuronal polarity formation by inhibitory neurons. These neurons are generated in the subpallium and migrate to the cortex. There they reach the marginal zone and execute multidirectional tangential migration, exhibiting a bipolar shape with a leading and trailing process. As development proceeds, they localize in the cortical plate, alternately extend and retract short processes, and exhibit low motility of somata. An axon then emerges (symmetry break) extending primarily toward the ventricle. Inhibitory neuron morphology is highly diverse: these subtypes include basket cells (the major inhibitory cortical neuron; dendrites, pink), Martinotti cells (the second major type; dendrites, yellow), double bouquet cells (the third major type; dendrites, blue) and others (Kubota, 2014). Whether dynamic process of axon formation depicted here correspond to all inhibitory neurons or subtypes of inhibitory neurons remains unknown.

Excitatory cortical neurons originate predominantly from radial glial progenitors in the cortical ventricular zone (VZ) (Figure 1). Asymmetric division of these cells generates both self-renewing progenitors and young neurons or intermediate progenitors, and those intermediate progenitors then further divide to increase neuronal number (Miyata et al., 2001, 2004; Noctor et al., 2001, 2004; Pontious et al., 2008). Newly-generated neurons migrate through the subventricular zone (SVZ) and intermediate zone (IZ) to reach the cortical plate (CP). There, later-generated neurons migrate past neurons generated earlier and eventually occupy more superficial positions, resulting in an inside-first/outside-last neurogenetic gradient (Angevine and Sidman, 1961). Following completion of this migration, these activities give rise to a sixed-layered cortical structure (Bayer and Altman, 1991).

Morphologically, bipolar progenitor cells are extraordinarily slender and extend a long apical process toward the pial surface and a short basal process toward the ventricle. During asymmetric cell division, the daughter cell destined to become a neuron assumes a multipolar shape from which emanates multiple short, thin processes in the SVZ/IZ (Tabata and Nakajima, 2003; Noctor et al., 2004). After repeated extension and retraction of processes over several hours, one process suddenly extends tangentially within the IZ (Hatanaka and Yamauchi, 2013; Namba et al., 2014; Sakakibara et al., 2014). That process continues to elongate and eventually becomes an axon, as indicated by its length, morphology, and accumulation axonal markers such as Kif5c560 in the tip (Hatanaka and Yamauchi, 2013; see also Section Intracellular Mechanisms). Immature axonal processes also contain bidirectional microtubule fibers (Sakakibara et al., 2014), and in this aspect exhibit the kind of mixed microtubule polarity typically seen in the trailing process of migrating cerebellar granule cells, which also becomes an axon (Rakic et al., 1996). The remaining short processes gradually transform into thick leading processes directed toward the pia (Hatanaka and Yamauchi, 2013; Sakakibara et al., 2014). By the time cell bodies reach the CP, they appear bipolar, extending a trailing process “behind” (opposite the direction of migration) and a leading process “in front” of the nucleus. Cells then migrate with glia-guided locomotion mode (Rakic, 1972; Nadarajah and Parnavelas, 2002) characterized by repeated short extensions and contractions of the leading process accompanied by saltatory cell movement. Once the leading process reaches the marginal zone (MZ), cells switch to somal translocation as their final mode of movement (Nadarajah and Parnavelas, 2002). The leading process then differentiates into a dendritic arbor-like structure (Hatanaka and Murakami, 2002), which probably develops into an apical dendrite. Since radial migration is accompanied by sustained trailing process elongation in the IZ (the future WM), this dynamic behavior eventually results in the typical pyramidal cell morphology in which an axon extends from the bottom of the soma toward the WM and an apical dendrite orients toward the pia. Thus, for excitatory cortical neurons, migration is closely related to establishment of prospective neuronal polarity.

### Development of inhibitory cortical neurons

Tangential migration of neurons that transgress the cortico-striatal boundary and enter the neocortex was first reported by de Carlos et al. (1996). Collectively, several subsequent studies using transplantation, genetic fate mapping, and cell labeling analysis *in vivo* and *in vitro* established that tangentially migrating neurons include inhibitory cortical neurons. Moreover, most, if not all, inhibitory cortical neurons in mouse are reportedly generated embryonically from regions in the subpallium, including the medial and caudal ganglionic eminences and the preoptic area (Gelman and Marin, 2010), although some investigators have called into question whether cells emerge from the preoptic area (Ceci et al., 2012). Inhibitory neurons from these regions are further subdivided into distinct morphological subtypes exhibiting specific axonal arbors and dendritic patterns. Each subtype displays a unique combination of neurochemical markers and firing properties (Gelman and Marin, 2010; Bartolini et al., 2013; Kubota, 2014). Although each inhibitory neuron subtype originates in a distinct region, their overall migration behavior appears similar: in general, immature neurons migrate tangentially over long distances toward the cortex (Nadarajah and Parnavelas, 2002; Tanaka et al., 2003; Lopez-Bendito et al., 2004). They enter the CP from the SVZ, pass through it, and reach the MZ (Tanaka et al., 2009), where they further execute multidirectional tangential migration and become dispersed throughout the cortex (Tanaka et al., 2006, 2009; Inada et al., 2011; Yanagida et al., 2012). In mouse, these neurons settle into their final positions in the CP postnatally (Hevner et al., 2004; Tanaka et al., 2009).

During tangential migration, inhibitory neurons exhibit a bipolar shape with either an unbranched or branched leading process and a short trailing process. Currently, knowledge of dynamic developmental processes of these neurons is limited. However, Yamasaki et al. (2010) used electroporation with *gfp* or *DsRed* plasmid to label cells in the medial ganglionic eminence at E12.5 to assess morphological changes in mouse inhibitory neurons. Perinatally, as those labeled neurons moved from the MZ to CP, they appeared to transform into a multipolar, “sea urchin”-like shape, exhibiting multiple, thin processes (Yamasaki et al., 2010); no further information relevant to cellular dynamics during this transition is yet available. These processes repeatedly extended and retracted for several hours until one process became unusually long; most of these long processes extended toward the WM, while a minority extended toward the pia. Based on elongation, length and growth dynamics, it is likely that all of these processes represent prospective axons. Currently, it is not known what kind of inhibitory neuron these cells differentiate into; however, the medial ganglionic eminence can produce a variety of inhibitory neurons including Martinotti cells, which extend an axon oriented perpendicular to the pial surface (Gelman and Marin, 2010; Kubota, 2014). The remaining processes of multipolar cells likely become dendrites; however, details of their maturation remain to be elucidated.

It remains uncertain whether these behaviors of inhibitory neurons originating in the medial ganglionic eminence at E12.5 occur in all inhibitory cortical neurons. Nonetheless, these analyses indicate that a subset of inhibitory neurons initiates their polarization from a multipolar cell stage. Future analysis using tools such as genetically-modified mice expressing subtype-specific markers or Cre/CreER drivers (Taniguchi et al., 2011) should determine whether polarity can be established via alternate mechanisms.

## Cortical neurons *in situ* and hippocampal neurons in dissociation culture show similar polarity formation

The morphological dynamics of neurons undergoing polarization *in vitro* has been well-studied using time-lapse imaging of hippocampal neurons in dissociated culture (Dotti et al., 1988). After plating, hippocampal neurons typically develop axons and dendrites in five stages (Figure 1): (1) initially round cells form filopodia and lamellipodia; (2) cells extend and retract multiple minor processes; (3) one process transforms into an axon; (4) that axon extends and remaining processes differentiate into dendrites; and (5) differentiated processes mature. The first three stages in particular are key to establishment of polarity.

Evidence indicates that the initial minor processes of cells grown in culture have equal potential to differentiate into an axon: for example, if one experimentally cuts off a process that has grown longer than the others (presumably the future axon), a new potential axon emerges *de novo* (Dotti and Banker, 1987). Thus, polarity can be definitively established after an axon becomes apparently stable. However, some neurons *in vivo* are polarized at the time of generation and likely retain some aspects apico-basal polarity of progenitors in the neuroepithelum, or they have front-rear polarity of migrating immature neurons (Hatanaka et al., 2012). Therefore, these neurons *in vivo* do not need to redefine polarity but rather can inherit aspects of polarity. Indeed, retinal ganglion cells (Zolessi et al., 2006; Randlett et al., 2011) and bipolar cells (Morgan et al., 2006) appear to inherit apicobasal polarity of their progenitors. In retinal ganglion cells, not only the appearance but also the polarized distribution of intracellular components, such as the centrosome and Golgi apparatus, exhibit inheritance of polarity during axon formation (Zolessi et al., 2006; Randlett et al., 2011). Migrating neurons use polarized cellular components to form a leading and a trailing process required for directed movement (Evsyukova et al., 2013). Currently, there are no imaging studies *in vivo* or *in situ* that directly demonstrate the dynamics of these components during axon formation of migrating neurons. Nonetheless, pontine nucleus neurons form an axon from their leading process (Kawauchi et al., 2006; Watanabe and Murakami, 2009; Shinohara et al., 2013), and trailing processes of cerebellar granule neurons transform into axons (Komuro et al., 2001), indicating that these neurons inherit some elements of front/rear (leading/trailing process) polarity. These mechanisms differ from polarity formation seen in dissociated hippocampal neurons, in which an axon emerges *de novo* from non-polarized cells.

In contrast, as described above, in both excitatory and inhibitory cortical neurons *in vivo* or at least *in situ*, polarity formation primarily occurs in multipolar cells in a manner similar to that seen in dissociated hippocampal neurons. In the next section, we focus on early polarity events that occur during multipolar cell stages prior to axon formation.

### Dynamic processes of polarity formation in excitatory cortical neurons

Radial glial cells, the main progenitor population of excitatory cortical neurons, are neuroepithelial cells that exhibit apicobasal polarity. However, young neurons and intermediate progenitors appear to lose that polarity by retracting apical and basal processes during asymmetric cell division and assuming a multipolar shape (Tabata and Nakajima, 2003; Noctor et al., 2004; Hatanaka and Yamauchi, 2013). Furthermore, intermediate progenitors retract all visible processes and round up prior to division (Miyata et al., 2004; Noctor et al., 2004, 2008), suggesting that they do not inherit the apicobasal polarity exhibited by their progenitors. In addition, multipolar cells do not exhibit stable front-rear polarity, which is seen in actively migrating cells, but instead show highly dynamic behavior, alternately extending and retracting multiple short processes usually <50 μm in length. These cells also show unsteady somal movement (Tabata and Nakajima, 2003; Sakakibara et al., 2014), and some apparently form transient thick processes used to change migration direction (Sakakibara et al., 2014). Random distribution of the centrosome in multipolar cells reported in an imaging study (Sakakibara et al., 2014) and in fixed preparations (Shoukimas and Hinds, 1978) also support the idea that polarity is undetermined at these neuronal stages. After a prolonged period of this activity, a new thin process suddenly emerges and elongates tangentially. Occasionally, a cell retracts that process, even after it reaches >50 μm, and extends another (Hatanaka and Yamauchi, 2013), an activity also observed in hippocampal neurons prior to polarity establishment (Dotti et al., 1988). Once a process exceeds 100 μm, it will likely become an axon and continue to elongate (Hatanaka and Yamauchi, 2013; Namba et al., 2014; Sakakibara et al., 2014). Thus, axon formation of excitatory neurons mostly occurs during the multipolar period while exhibiting unstable or fluctuating polarity.

Recently, several papers have reported that signaling and cytoskeletal proteins function in the multipolar-bipolar transition of excitatory neurons in the IZ (reviewed in Cooper, 2014). To further understand mechanisms governing axon formation in these neurons, it will be important to determine if loss-of-function of those factors merely locks neurons into a multipolar state or also prevents them from forming an axon.

### Dynamic processes of polarity formation in inhibitory cortical neurons

During multidirectional tangential migration in the MZ, inhibitory cortical neurons extend a leading process in the direction of their movement (Tanaka et al., 2009; Inada et al., 2011; Yanagida et al., 2012). After long periods of migration in the MZ (estimated >1d; Tanaka et al., 2009), these neurons descend to the CP (Tanaka et al., 2009). Concomitantly, many transform into multipolar cells that extend numerous short processes, most <50 μm in length, although some are longer (Yamasaki et al., 2010). Because cells in the multipolar stage do not translocate (that is, their soma does not change position significantly), they appear to terminate their migration and lose front-rear polarity. Their short processes repeatedly extend and retract and show no preferential direction of extension. After a prolonged period of this activity, one process abruptly elongates (initial axon formation). As observed in dissociated hippocampal neurons in culture and in excitatory neurons *in situ*, other processes occasionally extend up to 150–200 μm but fail to extend further, and eventually only one exceeds 200 μm in length and differentiates into an axon (Yamasaki et al., 2010). Thus, at multipolar stages inhibitory neurons likely do not have fixed polarity, and axon formation occurs in these cells *de novo*. Further study examining dynamic movement of cellular components in multipolar cells during axon formation should validate this view.

### Modes of polarity establishment

There are minor differences between behavior of cortical neurons *in situ* and hippocampal neurons *in vitro*. Although in both cases neurons initially appear multipolar, the mode of “random growth and retraction” of processes differs slightly. First, minor processes of hippocampal neurons *in vitro* show alternate increases and decreases in length, while those of cortical neurons *in situ* often show alternate appearance and disappearance of processes. Therefore, potential sites of axon initiation seem to be set at the very beginning of the polarization process *in vitro*. Second, the location of a hippocampal neuron cell body *in vitro* appears fixed during the multipolar stage, while that of excitatory or inhibitory cortical neurons *in situ* does not. These activities may be due to microenvironmental differences, such as adhesive properties: hippocampal neurons interact with a positively-charged planar substrate, while cortical neurons do not. Some of the activities one sees in hippocampal neurons in *in vitro* might be artifacts.

### Centrosome positioning during polarity formation

*In vitro* and *in vivo* studies suggest an instructive role for centrosome positioning in axon specification (Lefcort and Bentley, 1989; Zmuda and Rivas, 1998; de Anda et al., 2005, 2010; Andersen and Halloran, 2012). However, recent time-lapse observations of centrosomes in polarizing excitatory cortical neurons *in situ* reveal that different mechanisms may govern axon formation in these cells (Sakakibara et al., 2014; reviewed in Sakakibara et al., 2013). The centrosome tends to move toward the most actively growing process (the so-called “dominant process”) and that the initiating axon does not always behave as the dominant process. Neurons undergoing multipolar migration in the IZ form an axon by extending a dominant process toward which the centrosome orients. Thus, the centrosome positions at the base of initiating axon (Sakakibara et al., 2014). Similarly, in polarizing hippocampal neurons *in vitro*, one minor process becomes an axon and then behaves as the dominant process. In both cases, the centrosome attracted to the growing axon. On the other hand, neurons in the CP at later migration stages exhibit a leading process oriented toward the brain surface, which then behave as the dominant process and attract the centrosome. When an axon forms at the rear of these cells *in situ*, the centrosome does not translocate toward the initiating axon but rather remains oriented toward the leading process (Sakakibara et al., 2014). Although the latter mode of axon formation may not be primarily observed *in vivo*, these observations suggest that centrosome positioning is passively controlled by a balance of protrusive activities among processes and does not play an instructive role in excitatory cortical neurons *in vivo*. In migrating inhibitory cortical neurons, the primary cilium, whose basal body is formed from a centriole, reportedly regulates Sonic hedgehog-mediated reorientation of the leading process (Baudoin et al., 2012). However, the function of the primary cilium in extracellular cue-oriented axonogenesis in these neurons remains unclear. Clarification of the contribution of centrosome/primary cilium to neuronal polarization *in vivo* may further prompt our understanding of microtubule function underlying axonal morphogenesis.

## Cellular mechanisms underlying neuronal polarization

**Figure 2:**
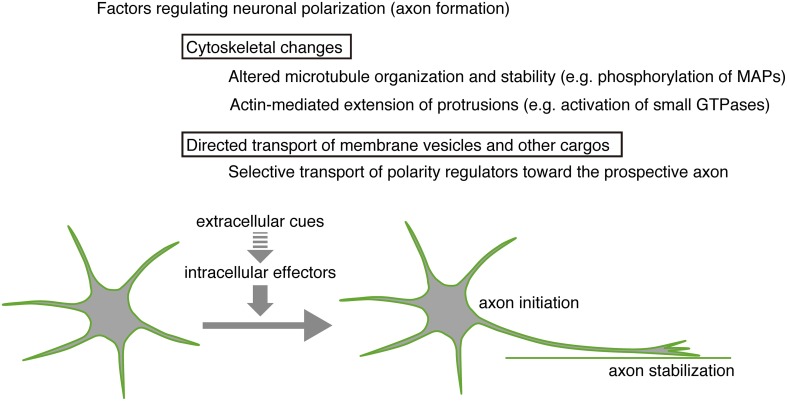
**Regulation of cortical neuron polarization.** Axon formation *in vivo* is influenced by extracellular cues. Intracellular effectors regulating cytoskeletal dynamics and membrane vesicle transport function in the initiation, stabilization, and subsequent elongation of a single axon.

Axon specification in cortical neurons is driven by intracellular and extracellular mechanisms (Figure 2). Intracellular signaling molecules relevant to polarization have been identified primarily in *in vitro* studies of hippocampal neurons, although *in vivo* studies validating these findings have also been reported. Extracellular mechanisms regulating cortical neuron polarization have been studied by *in vivo* analyses of knockout phenotypes or gene manipulation in embryonic mouse brain, although most of these studies have been confined to excitatory cortical neurons.

### Intracellular mechanisms

Cytoskeletal changes dependent on protein phosphorylation are required for axon specification; thus, axon formation can be assessed using markers recognizing differential phosphorylation states of cytoskeletal proteins (Sternberger and Sternberger, 1983; Mandell and Banker, 1996). Stable microtubules within axons confer distinct characteristics based on their organization (Witte et al., 2008; Conde and Cáceres, 2009), and signaling molecules like LKB1 and SAD-A/B kinases trigger axonogenesis by changing the phosphorylation state of microtubule-associated proteins (MAPs), such as Tau and DCX (Kishi et al., 2005; Barnes et al., 2007; Shelly et al., 2007). In excitatory cortical neurons, PKA reportedly activates LKB1, leading to phosphorylation of SAD kinases. Consequent downstream signaling of SAD kinases phosphorylates Tau at S262, an event thought to initiate axon formation (Kishi et al., 2005).

The aPKC/Par complex plays a central role in axon specification: for example, aPKC inhibition suppresses axon formation in hippocampal neurons (Zhang et al., 2007). aPKC/Par complex function is differentially regulated by Par3 phosphorylation via multiple kinase pathways (Funahashi et al., 2013; Yang et al., 2014). TGF-ß signaling reportedly increases Par6 phosphorylation, which is required for axon formation by excitatory cortical neurons (Yi et al., 2010). MARKs/Par-1, which acts downstream of the aPKC/Par complex, controls microtubule-binding affinity of DCX (Sapir et al., 2008). The DLK-JNK pathway also regulates DCX phosphorylation and that of other MAPs as well as SCG10/stathmin-2 (Gdalyahu et al., 2004; Eto et al., 2010; Hirai et al., 2011; Westerlund et al., 2011). DOCK7 activation of the small GTPase Rac controls MT stability in axons by inactivation of stathmin/Op18 (Watabe-Uchida et al., 2006). Thus, concerted regulation of microtubule function by multiple kinases and their effectors likely underlies axon formation.

Several small GTPases function differentially in neuronal polarization (Arimura and Kaibuchi, 2007; Gonzalez-Billault et al., 2012). Local activation of the Rap1-Cdc42 pathway has been observed at the tip of an initiating axon in hippocampal neurons *in vitro* (Schwamborn and Püschel, 2004). Cdc42 reportedly remodels the actin cytoskeleton via cofilin phosphorylation (Garvalov et al., 2007). Rac/Cdc42 also controls retrograde movement of F-actin via phosphorylation of downstream effectors such as PAK1 and Shootin1 (Toriyama et al., 2013). Interestingly, Shootin1 is implicated in potential crosstalk between the L1-cell adhesion molecule, F-actin, and microtubules in regulating growth cone dynamics during neuronal polarization (Shimada et al., 2008; Sapir et al., 2013), suggesting that coordinated regulation of actin and microtubules is critical for axon formation.

Axon formation also requires directed transport of membrane vesicles and other cargos along polarized microtubules (reviewed in Conde and Cáceres, 2009; Hirokawa et al., 2010; Stiess and Bradke, 2011; Sakakibara et al., 2013). Polarized transport by plus-end-directed motors, such as kinesin-1 (KIF5) and kinesin-2 (KIF3), plays a central role in establishing a single axon (Nakata and Hirokawa, 2003; Jacobson et al., 2006). Identification of several cargo molecules suggests that directed accumulation of signaling and scaffold proteins, such as CRMP-2, PAR-3, and JIP1, is important for axon specification (Nishimura et al., 2004; Kimura et al., 2005; Dajas-Bailador et al., 2008). Localized acetylation of microtubules may regulate cargo/microtubule affinity during axon specification (Reed et al., 2006). PIP_3_ transport by GAKIN/KIF13B functions in axon formation, suggesting a role for accumulated PIP_3_ in positive feedback regulation of Rac and Cdc42 small GTPases (Horiguchi et al., 2006). Shootin1 also is known as a cargo of Kif20b (Sapir et al., 2013).

Recent studies show that regulators of microtubule dynamics are required for neuronal polarization. Control of microtubule minus-end dynamics by CAMSAP2 is essential for polarization of cortical neurons *in vivo* (Yau et al., 2014). Altered plus-end dynamics induced by depletion of microtubule regulators, such as, SLAIN1/2, chTOG/XMAP215, and CLASP2, reportedly underlie polarization defects (Beffert et al., 2012; van der Vaart et al., 2012). These observations suggest that properly controlled microtubule growth, which also underlies microtubule-dependent directional transport, is essential to shape axons.

### Extracellular mechanisms

Interestingly, when the growth cone of an immature process of a hippocampal neuron encounters a preferred substrate *in vitro*, its growth rapidly increases, while that of other immature processes does not (Esch et al., 1999; Shelly et al., 2007). Observations such as this indicate that external cues also govern polarity formation. Contact with laminin in the basal lamina also triggers retinal ganglion cell axon formation (Zolessi et al., 2006; Randlett et al., 2011). In the case of excitatory cortical neurons, integrity of the surrounding microenvironment may greatly impact axonal specification. Indeed, recent work reveals that these neurons establish directed tangential axon outgrowth due to instructive cues presented on pre-existing efferents: close contact with the cell adhesion molecule TAG-1 on these efferents in the lower IZ stimulates axon formation by multipolar cells, an event mediated in part by downstream Lyn-kinase (Namba et al., 2014). In addition, several extracellular factors, such as the homotypic cell adhesion protein N-cadherin (Gärtner et al., 2012), diffusible protein TGF-ß (Yi et al., 2010) and neurotrophins (Nakamuta et al., 2011), reportedly function as polarization signals for excitatory cortical neurons *in vivo*. Some investigators propose that a single axon is specified via positive feedback signals that stabilize process extension (Arimura and Kaibuchi, 2007; Inagaki et al., 2011), suggesting that external cues, either contact-mediated or locally diffused, have a stabilizing effect on polarity. In the case of inhibitory neurons, the surrounding environment indeed appears to influence polarity formation: inhibitory neurons do not assume a multipolar shape in dissociated culture. Instead, one of the two processes emerging from these neurons elongates and eventually becomes an axon (Hayashi et al., 2003). Possible cell-cell interactions, such as tiling interactions between neighboring inhibitory neurons, might partially contribute to shape cells *in vivo*. Because excitatory cortical neurons under the same conditions appear multipolar (Hayashi et al., 2003), intrinsic mechanisms governing axon formation in these two types of neurons may differ despite their similar behavior *in vivo*.

## Concluding remarks

Here, we have reviewed recent evidence suggesting that cortical neurons, both excitatory and inhibitory, establish polarity *de novo*. These neurons initiate axons after assuming a multipolar stage, in which no fixed polarity is exhibited. Although these neurons appear similar during axon formation, it is important, especially in the case of inhibitory neurons, to examine the dynamics of cellular components to validate this view. Also, it will be interesting to examine whether these neuronal subtypes share signaling pathways governing polarity. It should be noted that inhibitory cortical neurons are in fact diverse and consist of multiple morphological subtypes with different spatial and temporal origins. Thus, future investigations are needed to determine whether these subtypes share a common mechanism of axon initiation. In addition, it is important to define dynamic processes governing dendrite formation by both excitatory and inhibitory cortical neurons. These analyses will likely require a combination of genetic labeling of specific excitatory and inhibitory neuronal subtypes with live imaging both *in situ* and *in vivo*.

Finally, analysis of dissociated hippocampal neurons has set the foundation for our current understanding of polarization processes and their molecular basis. Although some of this knowledge is applicable to cortical neurons *in situ*, care should be taken in generalizing these mechanisms to other neuronal types. Proper understanding of polarity formation in the cerebral cortex requires identification of the key processes that underlie external cue-mediated polarization *in vivo*.

### Conflict of interest statement

The authors declare that the research was conducted in the absence of any commercial or financial relationships that could be construed as a potential conflict of interest.
